# Comparative histopathological and immunohistochemical features of central nervous system and pulmonary tuberculosis: an exploratory retrospective study

**DOI:** 10.3389/fcimb.2026.1933605

**Published:** 2026-10-05

**Authors:** Kaili Su, Junjun Liu, Runrui Wu, Wen Chen, Yuejiao Liu, Yadong Liu, Xu Zhang, Min Fan, Libao Feng, Shuangshuang Zhao

**Affiliations:** 1 Hohhot Hospital of Traditional Chinese Medicine and Mongolian Medicine, Hohhot, China; 2 Eighth Medical Center of the Chinese PLA General Hospital, Beijing, China; 3 Henan University of Chinese Medicine, Zhengzhou, China

**Keywords:** central nervous system tuberculosis, drug resistance, granulomatous inflammation, immune cell, immunohistochemical (IHC)

## Abstract

Central nervous system tuberculosis (CNS-TB) is a rare and particularly severe form of extrapulmonary tuberculosis (EPTB). Early diagnosis and precise treatment are crucial in managing this condition. Pathological and immunohistochemical features may differ between CNS-TB and pulmonary tuberculosis (PTB). This study included 9 patients of CNS-TB, and analyzed their clinical manifestations, pathological features, and the immunohistochemical(IHC) features. The findings were then compared with PTB to explore the similarities and differences in how tuberculosis manifests in the brain versus the lungs. However, CNS-TB tissue specimens are difficult to obtain, limiting our sample size. Additionally, several patients received preoperative anti-tuberculosis therapy and short-course corticosteroids before sampling. Given these baseline differences, intergroup comparisons should be interpreted cautiously. Unlike patients with isolated PTB, those with CNS-TB frequently present with multisystem tuberculosis involving the lungs. Typical tuberculosis manifestations including fever, anorexia, weight loss, dizziness and lymphadenopathy did not differ between groups. Likewise, CBC parameters (hemoglobin, total white blood cell count, absolute neutrophil count and lymphocyte count) were comparable between CNS-TB and PTB groups. In this small cohort, general pathological features such as granulomas and caseous necrosis were similar in CNS-TB and PTB lesions. We further compared their IHC profiles. This exploratory analysis revealed that CNS-TB lesions harbored significantly fewer CD34^+^ microvessels, CD4^+^ T cells and CD38^+^ cells relative to PTB lesions, while CD56^+^ cells were mildly elevated in CNS-TB. No marked intergroup differences were noted for other immune cell subsets. Collectively, pathological characteristics and immune cell infiltration may differ between CNS-TB and PTB. Owing to the absence of normal brain and lung tissue controls, our findings warrant cautious interpretation. Finally, we performed exploratory drug-resistance analysis using remaining specimens from four patients in each group and observed similar resistance-associated mutation profiles between CNS-TB and PTB lesions.

## 1 Introduction

Tuberculosis(TB) is an infectious disease caused by the *Mycobacterium tuberculosis*(Mtb) complex. It leads to high incidence and mortality rates, especially in developing countries. Mtb typically affects the lungs, but it can also damage any organ in the body, leading to EPTB. Common EPTB in clinical practice include lymph node tuberculosis, osseous tuberculosis, and renal tuberculosis (Baykan et al., 2022). According to the World Health Organization(WHO), EPTB patients account for 15%–20% of all tuberculosis patients worldwide. Li found that in China, EPTB patients make up 21.3%, which is higher than the global proportion of EPTB among TB patients (16.0%) (Li et al., 2023). CNS-TB is a type of EPTB. It is primarily caused by Mtb, which initially infects the lungs, then spreads via the bloodstream to the brain and spinal cord, meninges, and surrounding tissues, where lesions develop. In clinical practice, It can be divided into three types: tuberculous meningitis, intracranial tuberculoma (tuberculous brain abscess), and spinal tuberculosis. CNS-TB often occurs secondary to tuberculosis in the lungs, lymph nodes, bones, and genitourinary system. Pulmonary tuberculosis is the most common form. The type of disease and the number of lesions depend on the number of Mtb, their virulence, and the host’s immune status.

Compared to other systems, the nervous system is less frequently affected by TB, but when it is, the condition is more severe. CNS-TB accounts for 1.5% to 3.2% of all tuberculosis-related deaths (Gupta and Kumar, 2011). The mortality rate among hospitalized patients is even higher, at 42.12% (Navarro-Flores et al., 2022). In terms of disability rates, CNS-TB ranks 11th, while its lethality rate ranks 13th (Murray et al., 2012).The clinical manifestations in patients include headache, irritability, vomiting, fever, nuchal rigidity, seizures, focal neurological deficits, and impaired consciousness. The prognosis of CNS-TB depends on the clinical stage of the disease and the patient’s age (Qureshi et al., 2002). Therefore, early diagnosis and treatment are crucial. The diagnosis of TB is confirmed through tests of local secretions, pathological staining for acid-fast bacilli, or PCR testing for Mtb. CNS-TB can also be diagnosed using imaging studies, especially MRI, which helps determine the location, size, and number of tuberculous lesions, thereby providing clues for diagnosis. However, since the clinical and imaging manifestations of CNS-TB are similar to those of other intracranial masses and infectious lesions, clinical symptoms are often atypical. Inflammatory exudates and pathogens are rarely found in living tissues (Filho et al., 2001). The positive rate of etiological tests is low. Additionally, the immune function of patients varies at different stages of the disease, and there is a lack of sensitive and effective detection methods. These factors make the diagnosis of CNS-TB very difficult.

Histopathological identification of granulomas with caseous necrosis is highly suggestive of TB. Definitive confirmation requires etiological evidence, such as positive Mtb culture or molecular testing for Mtb nucleic acids. The central nervous system is a complex, relatively closed structure composed of the brain and spinal cord, along with their unique blood-brain barrier and cerebrospinal fluid circulation pathways. The pathological conditions in CNS-TB are strictly regulated by the blood-brain barrier(BBB) and influenced by the complex neural tissue structure within the brain. Given the unique blood-brain barrier and structural characteristics of the central nervous system, differences in granuloma formation, inflammatory infiltration patterns, and pathogen spread between CNS-TB and PTB have been hypothesized. The immune-privileged microenvironment of the CNS may potentially modulate local immune activation and suppression, which might further influence pathological manifestations and treatment responses. These potential differences remain speculative and require further validation. Compared to PTB, CNS-TB presents challenges due to difficulties in sample collection and a lack of large patient populations. There is also limited reporting on differences in pathological characteristics between CNS-TB and PTB. Therefore, this study explored the differences between CNS-TB and PTB in terms of clinical presentation, pathological features, IHC features, and drug resistance, with the aim of providing a reference for early diagnosis and targeted treatment.

## 2 Materials and methods

### 2.1 Institutional case collection and sampling procedures

This study selected patients of CNS-TB diagnosed through histopathological examinations at the 8th Medical Centre of Chinese PLA General Hospital, from April 2014 to April 2025. All CNS-TB patients were surgical specimens. Inclusion criteria were: (1) age > 18 years; (2) tissue samples obtained via biopsy, puncture, or surgical resection, followed by pathological examination; (3) pathological confirmation based on positive molecular testing for Mtb; and (4) availability of archived paraffin blocks for further analysis. All patients underwent rapid plasma reagin testing and HIV screening, both of which were negative. Patients with concurrent malignant tumors, hematological malignancies, syphilis, AIDS, illicit drug use, or severe trauma were excluded.

All CNS-TB patients were confirmed by surgical specimens. Among the 9 CNS-TB patients, 7 received anti-tuberculosis treatment for 1–15 days before surgery, and 7 patients received a short course (1–10 days) of low-dose corticosteroid therapy before surgery. Peripheral blood samples for CBC were collected before surgical resection and before the initiation of corticosteroid therapy. Given that two patients were undergoing anti-tuberculosis treatment before blood sampling, we will treat hemoglobin, white blood cell count, absolute neutrophil count, and absolute lymphocyte count as binary variables. We will also include the timing of the blood test, as well as details regarding anti-tuberculosis treatment and hormone therapy, along with specific values and units, in the supplementary information. The control group comprised 9 PTB patients who were hospitalized during the same period and had a pathological diagnosis of PTB based on surgical specimens. Peripheral blood samples for CBC were collected before surgical resection, before the initiation of corticosteroid therapy and anti-tuberculosis treatment. The reference ranges for laboratory parameters were as follows: hemoglobin, 120–160 g/L for males and 110–150 g/L for females; total white blood cell count, 3.5–9.5×10^9^/L; absolute neutrophil count, 2.0–7.5×10^9^/L; absolute lymphocyte count, 0.8–4.0×10^9^/L.

### 2.2 H&E staining method

All samples to be examined were fixed in 10% formalin solution, routinely dehydrated, and embedded in paraffin. From each sample, a 3μm-thick section was cut. The sections were then dried at 72 °C for 30 minutes, treated with xylene for 10 minutes twice. They were subsequently exposed to ethanol gradients of 100%, 90%, and 80% for 5 minutes each. After washing, the sections were stained with hematoxylin for 2 minutes, then washed again. They were further treated with 1% hydrochloric acid solution, washed again, then counterstained with ammonia. After washing once more, the sections were stained with eosin for 5 seconds and washed again. Finally, the sections were dehydrated using ethanol gradients of 75%, 85%, 95%, and 100%, cleared with xylene, and mounted in neutral resin.

Histopathological features were defined according to standard diagnostic criteria. In this study, the following pathological terms were defined as: Granuloma: a focal collection of epithelioid cells, often with multinucleated Langhans giant cells and surrounding lymphocytes. Caseous necrosis: granulomatous lesion with central amorphous, eosinophilic, and structureless necrotic debris. Coagulative necrosis: tissue architecture largely preserved, with hypereosinophilic cells showing cytopathic changes, but without typical caseous necrosis. Abscess: a focal tissue space filled with dense, neutrophil-rich purulent exudate. Fibrous hyperplasia: proliferation of fibrous connective tissue, often observed as a reactive or reparative change in chronic inflammation. Acute inflammation: tissue infiltration predominantly composed of neutrophils. Inflammatory exudation: extravasation of protein-rich fluid and inflammatory cells within the interstitium or tissue spaces.

### 2.3 Acid-fast staining method

Prepare 3 tissue sections, each 3 μm thick, from the sample. After preparation, these sections are treated in a manner similar to the H&E staining procedure, up to the point of ethanol dehydration following washing. Next, 2–3 drops of carbol fuchsin stain are added and incubated at room temperature for 2–3 hours. After incubation, the sections are decolorized with 1% hydrochloric acid in ethanol until a light pink color is achieved. Subsequently, the sections are counterstained with hematoxylin once more. The sections are then differentiated using a 1% hydrochloric acid solution, followed by counterstaining with ammonia. Finally, the sections are dehydrated through gradients of 80%, 90%, and 100% ethanol, cleared in xylene, and mounted in neutral resin. Acid-fast staining reveals Mtb as red bacilli against a blue background.

### 2.4 *Mycobacterium* strain identification membrane strip method

To identify the mycobacterial species, 8–10 sections (5–10 μm thick) were cut from the archived paraffin blocks and placed into 1.5 mL centrifuge tubes. The sections were then deparaffinized, digested, and subjected to DNA extraction. For each sample, 4μL of extracted DNA was added to PCR tubes. PCR was performed on the fully automatic medical PCR analysis system (SLAN-96S; Shanghai Hengshi Medical Technology Co., Ltd.) using a mycobacterial species identification PCR kit (Shenzhen YaNeng Biotechnology Co., Ltd.). Positive and negative control DNA samples were processed simultaneously as quality controls.

PCR amplicons labeled with biotin are hybridized with probes immobilized on the membrane under specific temperature and salt concentration conditions. Biotin subsequently binds to streptavidin, and horseradish peroxidase (HRP) catalyzes the reaction to trigger color development of TMB.Amplification was carried out per the designated protocol: 2 min at 50 °C, 10 min at 95 °C, then 30 cycles (95 °C for 45 s, 68 °C for 60 s), and a final extension at 68 °C for 10 min. During hybridization, the membrane strip and amplicons were added to a tube with 5–6 mL Solution A, boiled for 10 min, and incubated at 59 °C for 1.5 h. MSI positive and negative controls were processed in parallel. After washing with Solution B and incubation in POD solution, the membrane strip was incubated in freshly prepared chromogenic solution for 10 min protected from light. Purified or deionized water was used to stop the reaction when blue bands/spots appeared on the membrane.

The presence or absence of color at a specific location on the membrane strip indicates whether the probe has hybridized with the DNA fragment, thereby identifying the bacterial species. For the MTC positive control, blue spots appeared at both the MTC probe site and the CC (control check) site, while no color was observed at other sites. For the negative control, color appeared only at the CC site. A valid test required color development at the CC site; absence of color at the CC site indicated an invalid assay, rather than a negative result. A sample was considered positive for Mtb when color was present at both the CC and MTC sites (**Figure 1**).

**Figure 1:**
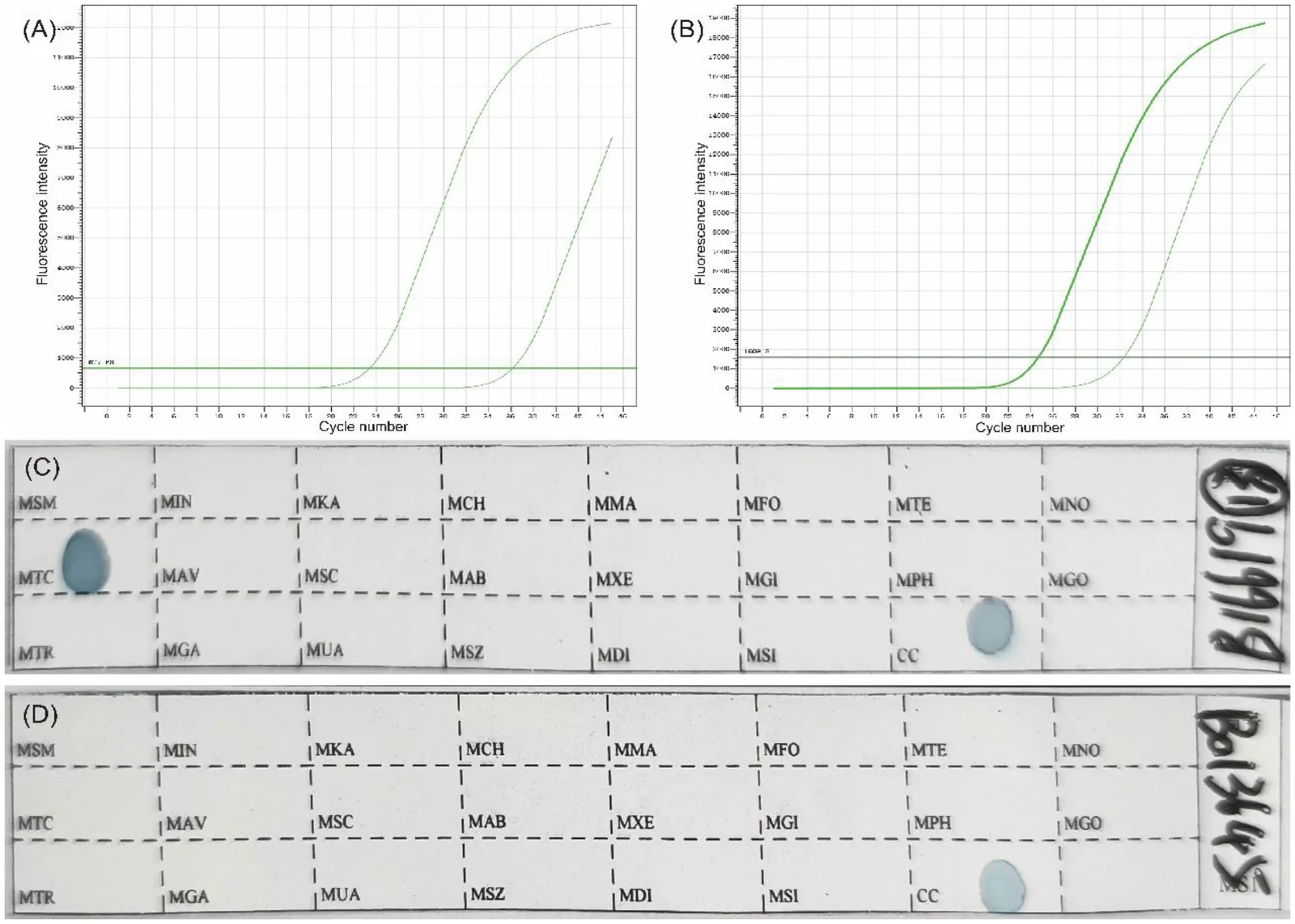
Real-time fluorescent quantitative PCR amplification curves. Amplification curves for PTB, CNS-TB samples, and positive controls **(A, B)**. The sequence of the detection site on the positive and negative membrane strip **(C, D)**.

### 2.5 Immunohistochemical staining

According to the requirements of the IHC staining procedure, 3μm-thick sections were cut from the paraffin-embedded tissue samples to be tested. The sections were dried at 55 °C overnight or at 60 °C for 1 hour. The sections were then placed in the appropriate positions on the Ventana Benchmark ULTRA staining platform. Reagents for the primary and secondary antibody assays, as well as amplification kits, hematoxylin, and counterstaining solutions were prepared and placed on the reagent rack. The entire IHC process was automated. During staining, negative and positive controls were used: known positive sections served as the positive control, while negative controls provided by Roche Diagnostics were used as the negative control. After the procedure, the sections were routinely dehydrated, cleared, and mounted.

PD-L1 interpretation criteria: The PD-L1 Immunocyte Proportion Score (IPS) represents the percentage of immunocytes that exhibit complete or partial membrane staining at any intensity. The Ki-67 proliferation index was categorized into three ordinal grades (low, intermediate, high) based on the proportion of Ki-67 positive cells among all evaluable cells within granulomatous lesions. The grading criteria were defined as follows: low proliferation, Ki-67 positive rate <10%; intermediate proliferation, Ki-67 positive rate 10%–30%; high proliferation, Ki-67 positive rate >30%. IHC staining for Ki-67 was performed, and at least 5 representative visual fields of granulomatous lesions were selected to calculate the percentage of Ki-67 positive nuclei. IHC staining was independently evaluated by two senior pathologists in a blinded manner. After excluding the central necrotic areas of lesions, five non-overlapping fields containing lesions and adjacent tissues were randomly selected under a 40× objective lens. The mean value was calculated based on manual cell counting.

The primary antibodies include Ki-67 (ZM-0166 mouse monoclonal antibody), CD3 (ZM-0417 mouse monoclonal antibody), CD4 (ZM-0418 mouse monoclonal antibody), CD8 (ZA-0508 rabbit monoclonal antibody), CD20 (ZM-0039 mouse monoclonal antibody), CD38 (ZM-0422 mouse monoclonal antibody), CD34 (ZM-0046 mouse monoclonal antibody), CD56 (ZM-0057 mouse monoclonal antibody), CD68 (ZM-0060 mouse monoclonal antibody), LCA (ZM-0183 mouse monoclonal antibody), and PD-1 (ZM-0381 mature monoclonal antibody). All of these are supplied by Beijing Zhongshan Jinqiao Biotechnology Co., Ltd. PD-L1 (SP263) and the secondary antibody detection system are provided by Roche Diagnostics.

### 2.6 Drug resistance of Mtb

Detection of Mtb drug resistance was performed using fluorescence PCR melting curve assay. Briefly, 8–10 tissue sections (5–10 μm thickness) were prepared. Each section was placed into a 1.5 mL centrifuge tube, followed by dewaxing, lysis, digestion and DNA extraction. PCR amplification was then carried out. A total of 25 μL sample DNA was added to PCR tubes using the Mtb drug resistance detection kit (Xiamen Zhishan Biotechnology Co., Ltd.). The amplification protocol adopted dual-channel targets for rifampicin (RFP), ethambutol (EMB), isoniazid (INH), and streptomycin (SM), and a single-channel target for fluoroquinolones (FQs). Results were analyzed after amplification. The kit employed fluorescence PCR melting curve analysis to detect resistance-associated genes of Mtb for each anti-tuberculosis drug to identify genotypic resistance.

The experimental principle was as follows: fluorescence and quencher probes labeled at opposite termini were added to the PCR reaction system. Sequences complementary to the probes were amplified during PCR. After amplification, melting curve analysis was performed with real-time fluorescence monitoring. The negative derivative of fluorescence against temperature was calculated to generate melting curves for probe-target hybrids, allowing determination of melting temperature (Tm) and Tm shifts relative to the positive control, which indicated sequence mutations.

When the target sequence perfectly matched the probe, the hybrid yielded the highest Tm. In contrast, target sequences carrying mismatches such as point mutations, insertions or deletions produced lower Tm values. The magnitude of Tm reduction depended on the mutation type, the number of inserted/deleted bases, and mutation location. This genotypic assay interrogated known resistance-associated loci: the *ahpC* promoter region (positions −44 to −40), *inhA* codon 94, the *inhA* promoter region (positions −17 to −8), and *katG* codon 315 for isoniazid resistance; *embB* codons 306, 406 and 497 for ethambutol resistance mutations; *gyrA* codons 88–94 for fluoroquinolone resistance mutations; and *rpoB* codons 507–533 for rifampicin resistance variants.

Samples were classified as wild-type if Tm values across all channels were consistent with the positive control (error ≤1°C). A sample was defined as mutant when the Tm in any channel was ≥2°C lower than the positive control, indicating genotypic drug resistance. Absence of signal in a single channel suggested gene deletion, and such samples were categorized as mutants. Lack of melting peaks in two or more channels indicated undetectable target DNA, which may result from the absence of Mtb, low Mtb load, template concentration below the kit limit of detection, experimental operation errors, or kit failure.

For single-channel fluoroquinolone detection, mixed populations (coexistence of wild-type and mutant sequences) were considered present under the following conditions: double peaks or fused peaks with a Tm matching the positive control but markedly altered peak shape; small peaks or shoulders at the expected mutation Tm position; or fused peaks at the mutation Tm accompanied by minor peaks/shoulders at the wild-type Tm position. Samples with mixed populations were interpreted as fluoroquinolone-resistant. Retesting was recommended to confirm resistance for samples showing mixed peaks.

Interpretation of the negative control: the negative control contained TB DNA extraction solution without template and was used to assess aerosol nucleic acid contamination during DNA extraction and sample preparation. A melting peak in the negative control indicated nucleic acid contamination in the experimental environment. Additionally, peak morphology assisted interpretation for samples with low template concentrations. No melting peak in the test sample suggested absence of valid target signal, possibly due to: (1) no Mtb or Mtb template concentration below the kit detection limit; (2) errors in DNA extraction and sample preparation. In such patients, DNA re-extraction and repeated testing were required. If no melting peak was observed for the positive control despite correct reagent handling and sample preparation, kit failure was suspected. Laboratory contamination, reagent contamination and sample cross-contamination could produce false-positive results. Improper reagent transportation, storage or preparation reduced assay performance and caused false-negative or inaccurate results.

### 2.7 Statistical analysis

Images were processed using the image processing software Adobe Illustrator 2023. Statistical analysis of the data was performed using SPSS 27. For comparing between different groups of categorical variables, the Fisher’s test was used. For continuous variables that follow a normal distribution, the independent samples t-test was employed. For continuous variables that do not follow a normal distribution, the Mann-Whitney U test was used. Results with a P-value of < 0.05 was considered statistically significant.

## 3 Results

### 3.1 Clinical features

There were 9 patients in the PTB group, with ages ranging from 43 to 75 years, and an average age of 57.56 years. The group consisted of 7 males and 2 females. Among the 9 patients of CNS-TB, there were 4 males and 5 females, with ages ranging from 27 to 49 years, and an average age of 41.22 years.

When comparing the general demographic characteristics of the two groups of patients, such as gender, age, and medical history, CNS-TB was associated with tuberculosis in multiple site, including the lungs. Of the 2 patients who had received anti-tuberculosis treatment prior to CBC, hemoglobin, leukocyte count, absolute neutrophil count, and absolute lymphocyte count were analyzed as categorical variables. There was no statistically significant difference in the severity of these results between patients with CNS-TB and those with PTB (**Table 1**).

**Table 1:** Comparison of clinical data between PTB and CNS-TB.

Characteristic	PTB	CNS-TB	P	FDR-adjusted P	Effect size	95%CI
	(n=9)	(n=9)				
Gender(male)	7(77.78%)	4(44.44%)	0.335	0.490	4.375	0.564~33.949
Age(years)	57.56	41.22				
Combining tuberculosis in other areas	0	5(55.55%)	0.029	0.136	2.250	1.084~4.671
Fever	3(33.33%)	7(77.78%)	0.153	0.323	0.143	0.018~1.161
Anorexia	0	1(11.11%)	1	1	/	/
Weight loss	1(11.11%)	3(33.33%)	0.576	0.684	0.250	0.021~3.041
Dizziness	0	2(22.22%)	0.471	0.597	/	/
Lymphadenopathy	0	2(22.22%)	0.471	0.597	/	/
Decline in hemoglobin	1	2(22.22%)	1	1	0.438	0.032~5.926
Abnormal leukocyte count	0	1(11.11%)	1	1	/	/
Abnormal absolute neutrophil count	1(11.11%)	1(11.11%)	1	1	1	0.053~18.915
Abnormal absolute lymphocyte count	0	1(11.11%)	1	1	/	/

The value outside the bracket represents the"number of patients", while the value inside the bracket represents the "percentage (%)".

### 3.2 Imaging findings

CT scans in PTB patients show nodular high-density lesions typical of pulmonary tuberculosis. Samples are obtained through surgical resection or lung tissue biopsy. In CNS-TB patients, MRI scans reveal mixed signal patterns, with nodular ring-shaped enhancements on enhanced MRI. Samples are obtained through surgical resection or lesion biopsy (**Figure 2**).

**Figure 2:**
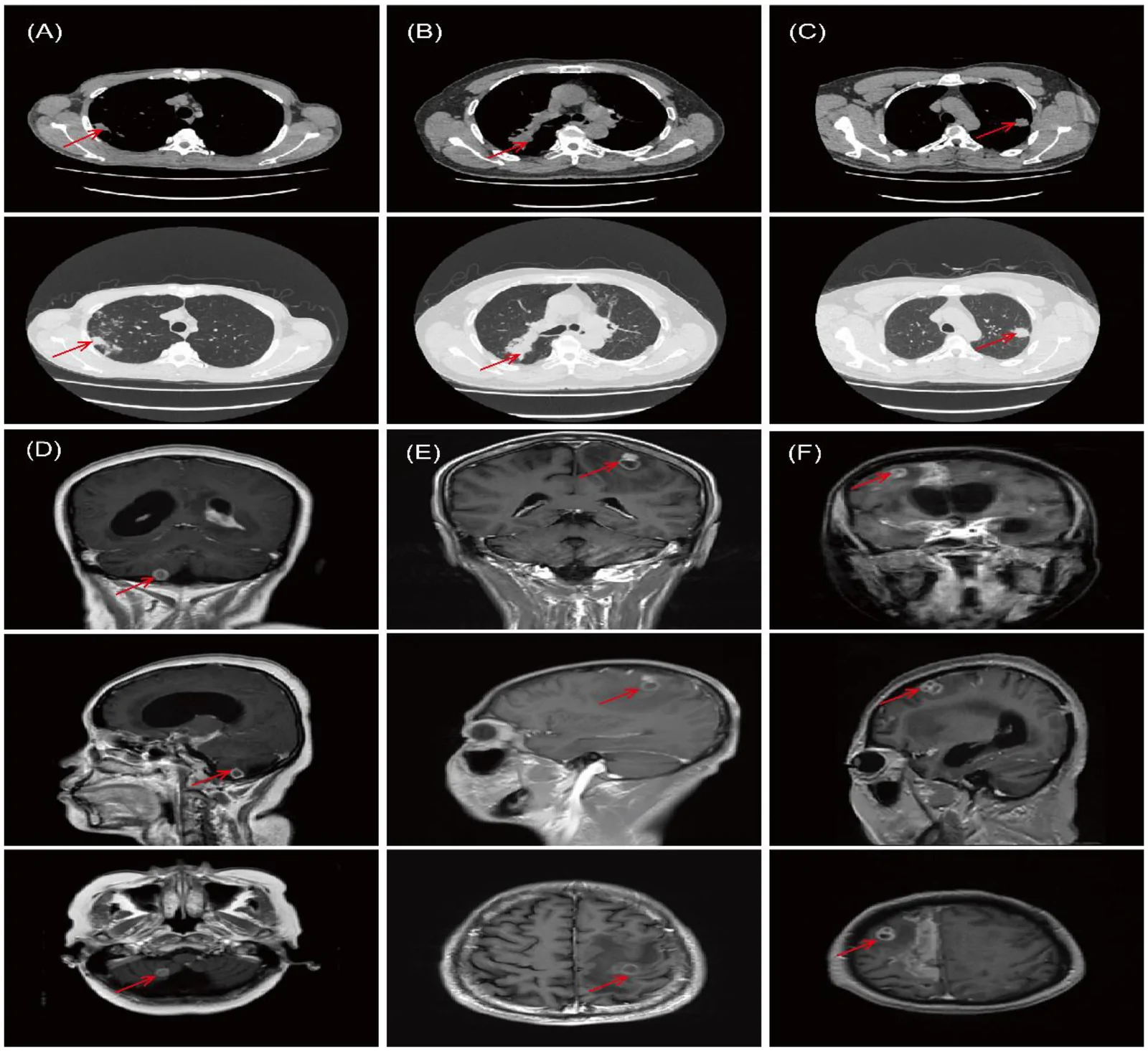
The lung CT shows scattered patchy, cord-like, and nodular high-density areas **(A)**. Multiple patchy high-density areas, nodules, and cord-like structures are visible on the lung CT, with some areas showing calcification **(B)**. Irregular mass-like lesions are also observed on the lung CT, with punctate dense areas inside. The edges of these lesions are rough, and they are partially lobulated, with traction on the pleura **(C)**. Post-contrast MRI shows nodular, ring-shaped enhancements in the cerebellar hemispheres **(D)**. In the left fronto-parietal junction area, oval-shaped mixed signals are observed. Large areas of slightly prolonged T1 and T2 signals are seen around these lesions **(E)**. The brain MRI also reveals scattered spots and nodular, ring-shaped enhancements. Irregular, atypical enhancements are observed in the right fronto-parietal lobe. Some areas of the meninges show varying degrees of enhancement **(F)**.

### 3.3 Histologic features

In the CNS-TB group, acid-fast staining was positive in 6 patients (66.67%). Granulomas and caseous necrosis were observed in 44.44% of patients (n=4) and 33.33% of patients (n=3), respectively. In the PTB group, 1 patient (11.11%) showed positive acid-fast staining. The incidences of granulomas and caseous necrosis were 77.78% (n=7) for both conditions. In this exploratory comparison, we did not detect obvious differences in general pathological features between CNS-TB and PTB lesions. Given the limited sample size, this finding should be interpreted cautiously. Although the positivity rate was notably higher in the CNS-TB group (6/9, 66.7%) than in the PTB group (1/9, 11.1%), the difference was only marginally significant (Fisher’s exact test, P = 0.05; 95% CI for the risk difference: 0.005 to 0.760), likely reflecting the limited statistical power of our small exploratory cohort.

The incidence of coagulative necrosis, abscesses, inflammatory exudation, fibrous thyperplasia, and acute inflammation in PTB patients was 11.11% (n=1), 11.11% (n=1), 100% (n=9), 66.67% (n=6)and 11.11% (n=1), respectively. In CNS-TB patients, the incidence of coagulative necrosis, abscesses, inflammatory exudation, fibrous tissue proliferation, and acute inflammation was 44.44%(n=4), 44.44% (n=4), 100% (n=9), 44.44% (n=4)and 55.55% (n=5) respectively. No statistical differences were observed between the two groups, though these exploratory comparisons are constrained by the small sample size (**Table 2**; **Figures 3**, **4**).

**Table 2:** Important pathological features between PTB and CNS-TB.

Characteristic	PTB	CNS-TB	*P*	FDR-adjusted *P*	Effect size	95%CI
	(n=9)	(n=9)				
Methods						
Acid-fast(+)	1(11.11%)	6(66.67%)	0.050	0.190	0.063	0.005~0.760
Pathology						
Granuloma	7(77.78%)	4(44.44%)	0.335	0.490	4.375	0.564~33.949
Caseous necrosis	7(77.78%)	3(33.33%)	0.153	0.323	7.000	0.861~56.895
Coagulative necrosis	1(11.11%)	4(44.44%)	0.294	0.490	0.156	0.013~1.828
Abscess	1(11.11%)	4(44.44%)	0.294	0.4890	0.156	0.013~1.828
Inflammatory exudation	9(100%)	9(100%)	1	1	/	/
Fibrous hyperplasia	6(66.67%)	4(44.44%)	0.673	0.752	2.500	0.370~16.888
Acute inflammation	1(11.11%)	5(55.55%)	0.131	0.323	0.100	0.009~1.170

The value outside the bracket represents the"number of patients", while the value inside the bracket represents the "percentage (%)".

**Figure 3:**
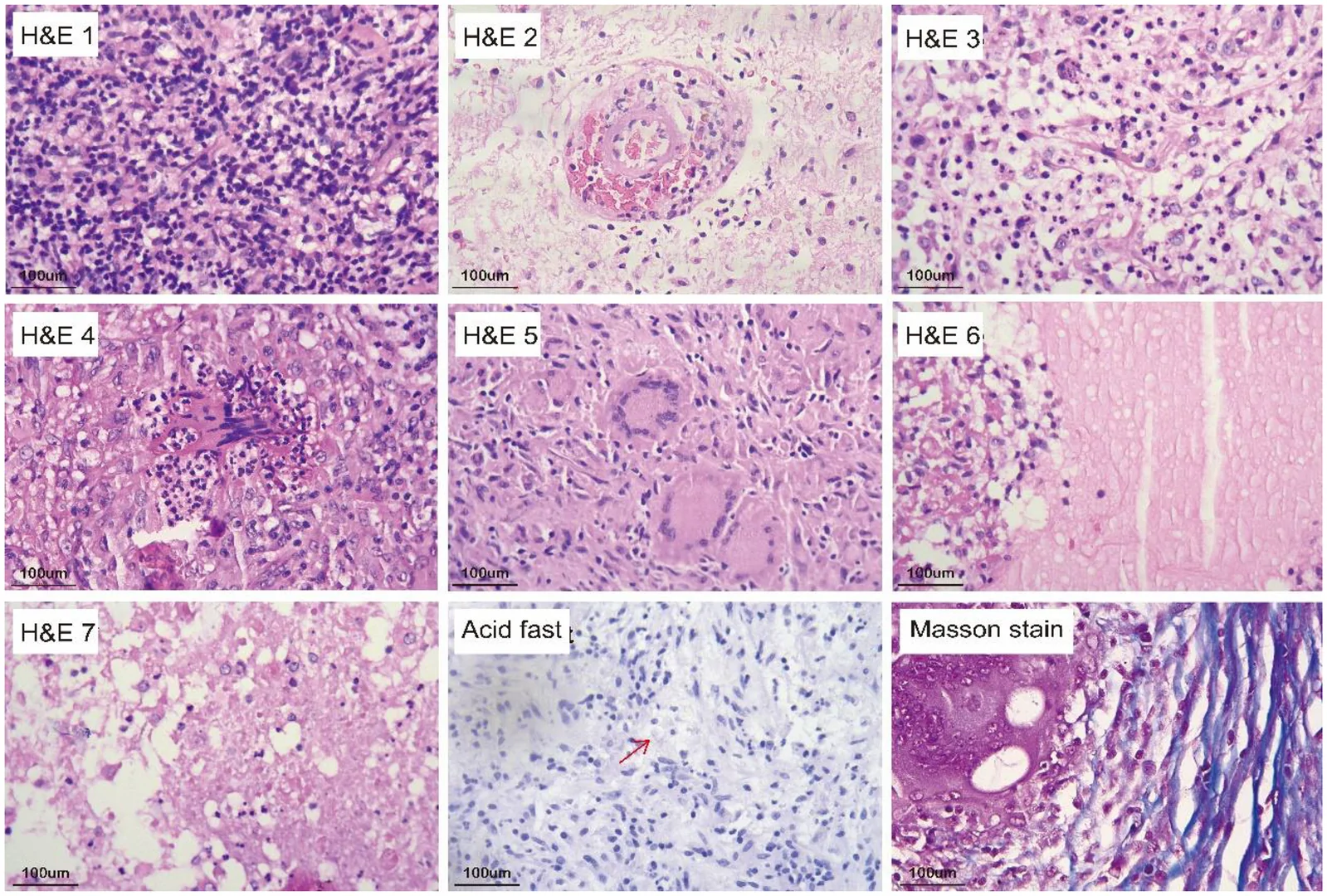
CNS-TB is characterized by chronic inflammation (H&E 1) and typical pathological features such as granulomas (H&E 5) with caseous necrosis (H&E 6).H&E staining reveals inflammatory exudate (H&E 2), acute inflammation (H&E 3), abscesses (H&E 4), and coagulative necrosis (H&E 7). Acid-fast staining reveals the presence of Mtb. Masson staining indicates fibrous proliferation. Scale: 100μm. Magnification of images: H&E staining (400x), acid-fast staining (400x), Masson’s staining (400x).

**Figure 4:**
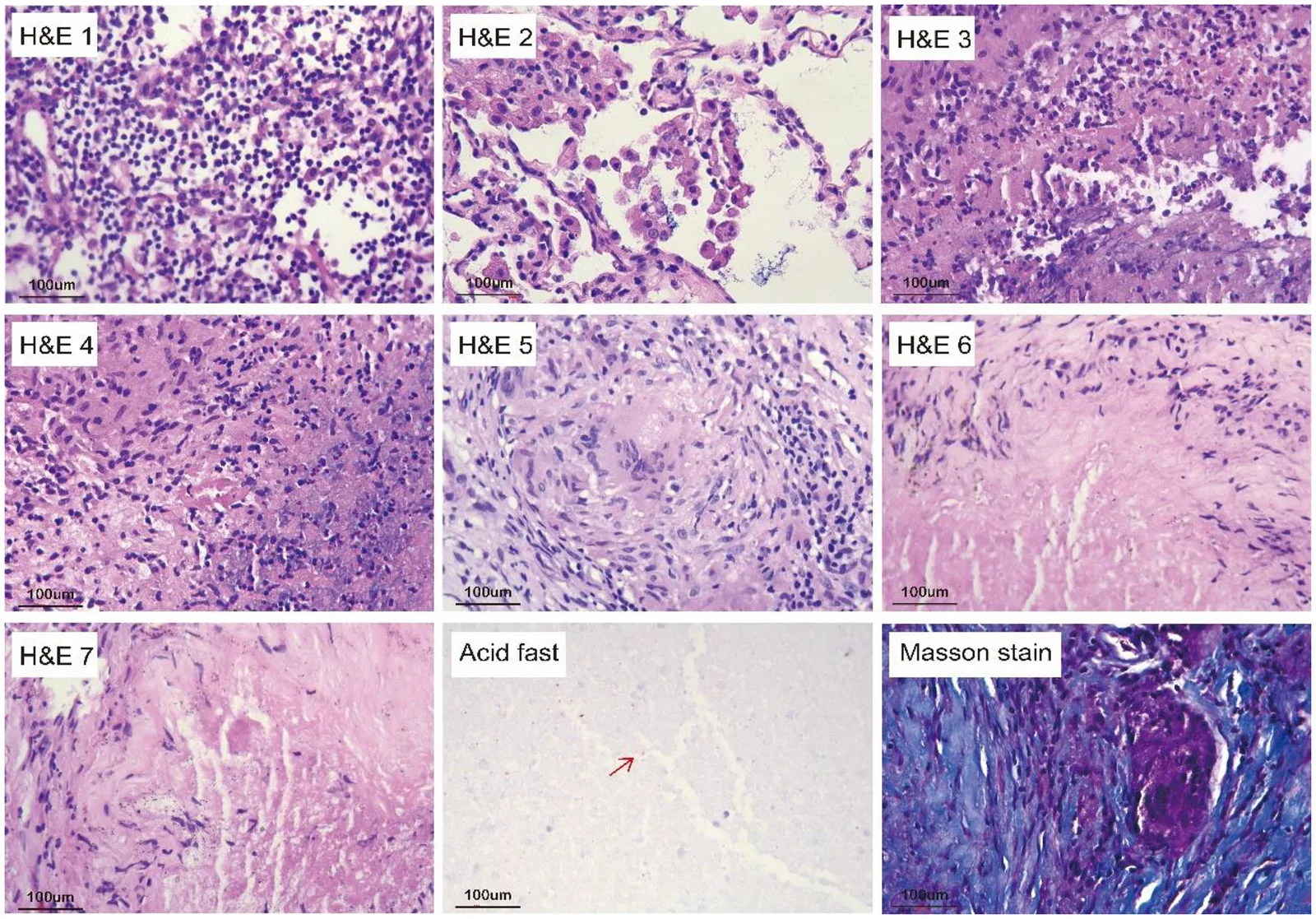
PTB is characterized by chronic inflammation (H&E 1) and typical pathological features such as granulomas (H&E 5) with caseous necrosis (H&E 6).H&E staining reveals inflammatory exudate (H&E 2), acute inflammation (H&E 3), abscesses (H&E 4), and coagulative necrosis (H&E 7). Acid-fast staining reveals the presence of Mtb. Masson staining indicates fibrous proliferation. Scale: 100μm. Magnification of images: H&E staining (400x), acid-fast staining (400x), Masson’s staining (400x).

### 3.4 Immunohistochemistry results

IHC staining showed that no marked intergroup differences were observed in the counts of CD3^+^ T cells, CD8^+^ T cells, CD68^+^ macrophages, LCA^+^ leukocytes and CD20^+^ B cells between the CNS-TB and PTB groups in this exploratory analysis. No significant differences were found in immune cell proliferation indices, as well as PD-1 and PD-L1, protein molecules closely related to immune cell function. However, compared with the PTB group, the CNS-TB group exhibited significantly lower counts of CD34^+^ vascular endothelial cells, CD4^+^ T cells and CD38^+^ cells with plasma-cell morphological features, whereas CD56^+^ infiltrating immune cells were more abundant in CNS-TB lesions, with statistically significant intergroup differences. This exploratory analysis is limited by the small sample size and imbalanced baseline clinical characteristics (**Table 3**; **Figures 5**, **6**).

**Table 3:** Comparison of immune cells in PTB and CNS-TB lesions.

Cell type	PTB	CNS-TB	F/U	P	FDR-adjusted P	Effect size	95% CI
	(n=9)	(n=9)					
Independent Samples t-test	Mean ± Standard Deviation		F			Cohen's d	
CD3^+^T-cells (cells/HPF)	401.78 ± 259.42	212.33 ± 154.97	1.637	0.078	0.203	-0.888	-1.603~-0.114
Microvessel (vessels/HPF)	22.44 ± 12.78	9.44 ± 5.90	3.427	0.004	0.091	-1.309	-2.080~-0.448
CD8^+^ T-cells (cells/HPF)	160.89 ± 94.09	105.89 ± 63.29	0.978	0.165	0.306	-0.687	-1.354~0.032
Macrophage (cells/HPF)	100.44 ± 57.21	87.00 ± 77.80	2.729	0.682	0.682	-0.198	-0.847~0.464
Leukocytes (cells/HPF)	91.33 ± 60.93	73.00 ± 59.56	0.142	0.528	0.596	-0.305	-0.963~0.373
Programmed Death-Ligand 1 (cells/HPF)	87.00 ± 51.40	90.33 ± 73.08	1.757	0.912	0.586	-0.288	-0.945~0.390
Mann-Whitney U Test	Median (P25,P75)		U			Cliff's δ	
CD4^+^ T-cells (cells/HPF)	236.00 (100.50,374.00)	88(7.50,146.50)	16.000	0.030	0.787	-0.540	-0.798~-0.094
B-Cells (cells/HPF)	44 (33.00,182.50)	28(4.50,63.00)	21.000	0.089	0	-0.431	-0.734~0.024
CD38^+^ cells (cells/HPF)	52 (35.00,74.50)	6(0.50,22.50)	4.000	<0.001	0.052	-0.806	-0.924~-0.440
CD56^+^ cells (cells/HPF)	0(0,1)	3(0.50,11.50)	15.000	0.022	0.787	-0.596	-0.823~-0.164
Programmed cell death receptor 1	0(0,0)	0(0,0)	38.500	0.718	0.863	-0.188	-0.562~0.257
Proliferation rate of immune cells	8 low, 1 moderate, 0 high	6 low, 3 moderate, 0 high	49.5	0.436	0.760	0	0

1. Data represent cell counts per high-power field (HPF, 40× objective).

2. For measurement data following a normal distribution, the mean ± standard deviation (x ± s) is used to represent the data. Comparisons between groups are performed using Independent-samples t-test.

3. For measurement data that do not follow a normal distribution, the median and interquartile range (IQR) are used for representation. Comparisons between groups are performed using the Mann-Whitney U test.

**Figure 5:**
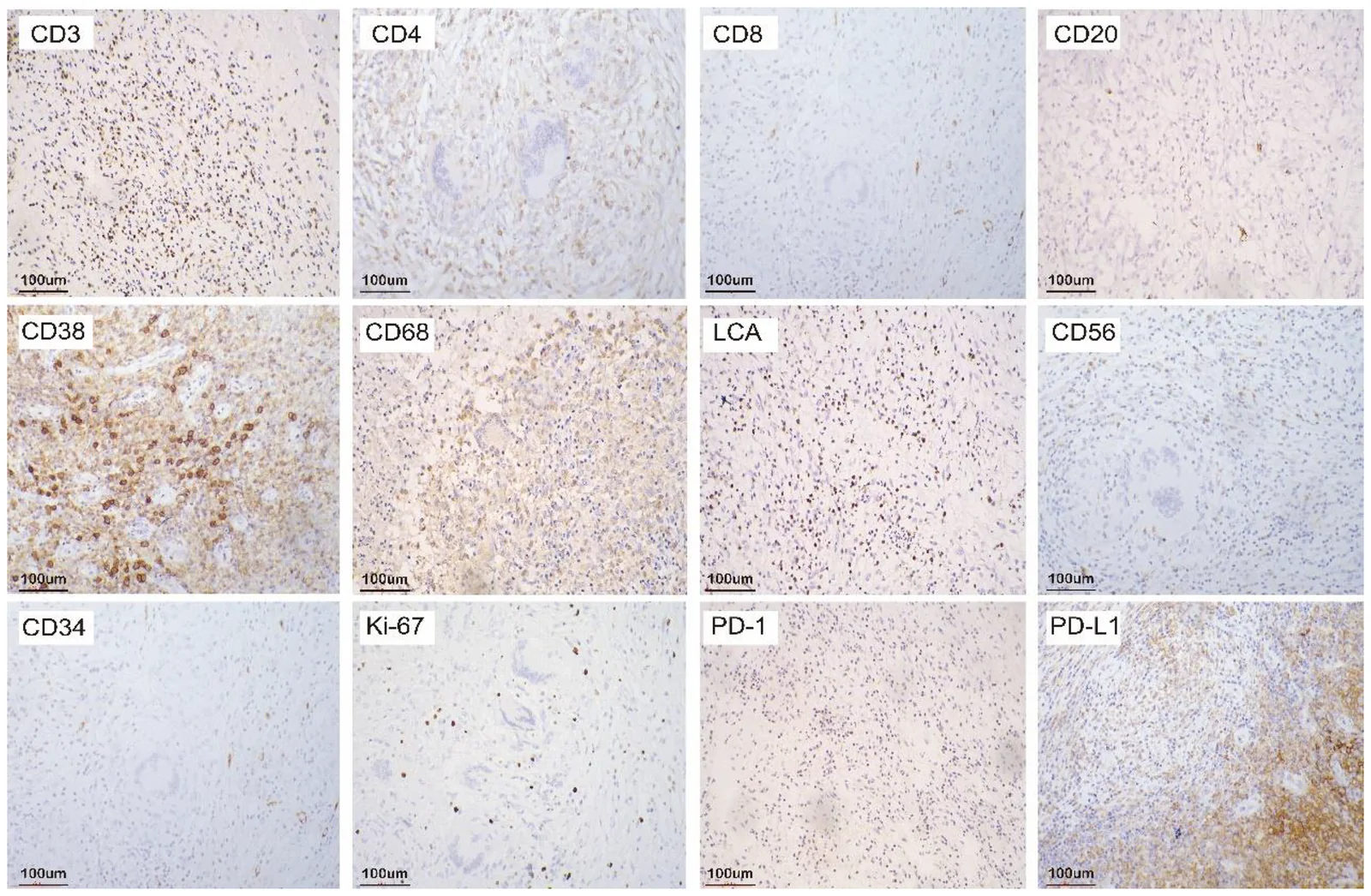
The immune microenvironment in CNS-TB lesions. Immunohistochemical staining shows labeled CD4^+^ T cells(CD4), microvessel(CD34), CD38^+^ cells (plasma-cell morphology), CD56^+^ cells (CD56), CD8^+^ T cells(CD8), Programmed Death-Ligand 1(PD-L1), CD3^+^ T cells(CD3), B cells (CD20), macrophages (CD68), leukocytes(LCA), programmed cell death receptor 1 (PD-1), and proliferation rate of immune cells(Ki-67). Scale: 100 μm; Magnification: Immunohistochemical staining (200x).

**Figure 6:**
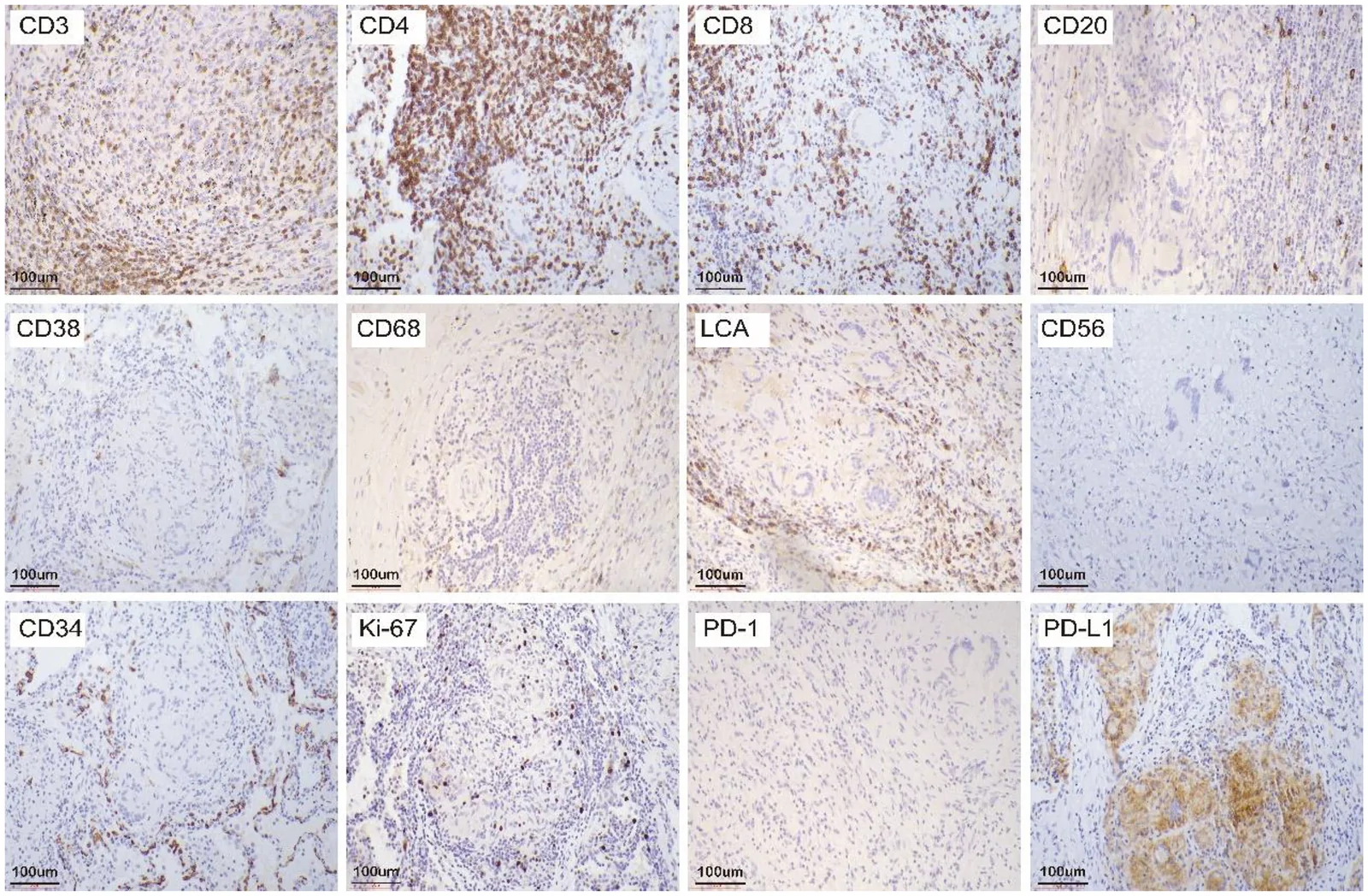
The immune microenvironment in PTB lesions. Immunohistochemical staining shows labeled CD4+ T cells(CD4), microvessel(CD34), CD38^+^ cells (plasma-cell morphology), CD56^+^ cells (CD56), CD8+ T cells(CD8), Programmed Death-Ligand 1(PD-L1), CD3+ T cells(CD3), B cells (CD20), macrophages (CD68), leukocytes(LCA), programmed cell death receptor 1 (PD-1), and proliferation rate of immune cells(Ki-67). Scale: 100 μm; Magnification: Immunohistochemical staining (200x).

### 3.5 Drug resistance results

Due to DNA degradation over time, the DNA content in some samples was insufficient for testing. As a result, 4 samples each from patients with nervous system tuberculosis and pulmonary tuberculosis were selected for tuberculosis drug resistance testing. The results showed that in both the CNS-TB and PTB groups, one patient in each group tested positive for a mutation related to fluoroquinolones. Furthermore, one patient in each group tested positive for mutations associated with resistance to both of the drugs. Research has revealed similarities in the resistance-related mutations found in CNS-TB and PTB. However, due to the small sample size and inconsistent sample sources, these preliminary findings are insufficient to support the inference that resistance patterns observed in PTB can be extrapolated to CNS-TB. (**Tables 4**, **5**; **Figure 7**).

**Table 4:** Detailed information on PTB and CNS-TB resistance.

Group	Case No.	Fluoroquinolones	Ethambutol	Streptomycin	Isoniazid	Rifampicin	Number of detected mutations
CNS-TB	1	+	+	+	+	−	4
	2	+	−	+	−	−	2
	3	+	−	−	−	−	1
	4	+	+	−	+	+	4
PTB	1	+	+	+	+	−	4
	2	+	−	−	−	−	1
	3	−	−	+	+	+	3
	4	+	−	+	+	−	2

"+" indicates the presence of a mutation related to drug resistance; "-" indicates the absence of a mutation related to drug resistance.

**Table 5:** Comparison of drug resistance.

Characteristic	PTB	CNS-TB	P	FDR-adjusted P	Effect size	95%CI
	(n=4)	(n=4)				
**Drug Resistance**	4(100%)	4(100%)	1	1	/	/
**Fully sensitive**	0	0	1	/	/	/
**Monoresistance**	1(25%)	1(25%)	1	1	/	/
Fluoroquinolones	1(25%)	1(25%)	1	1	/	/
**Multidrug resistance**	3(75%)	3(75%)	1	1	/	/
Dual drug resistance	1(25%)	1(25%)	1	1	/	/
Triple drug resistance	1(25%)	0	1	/	/	/
Four drug resistanc	1(25%)	2(50%)	1	1	/	/

The value outside the bracket represents the"number of patients", while the value inside the bracket represents the "percentage (%)".Bold rows denote the count of distinct drug resistance mutation categories. The subsequent non-bolded rows list the specific drugs associated with these resistance mutations.

**Figure 7:**
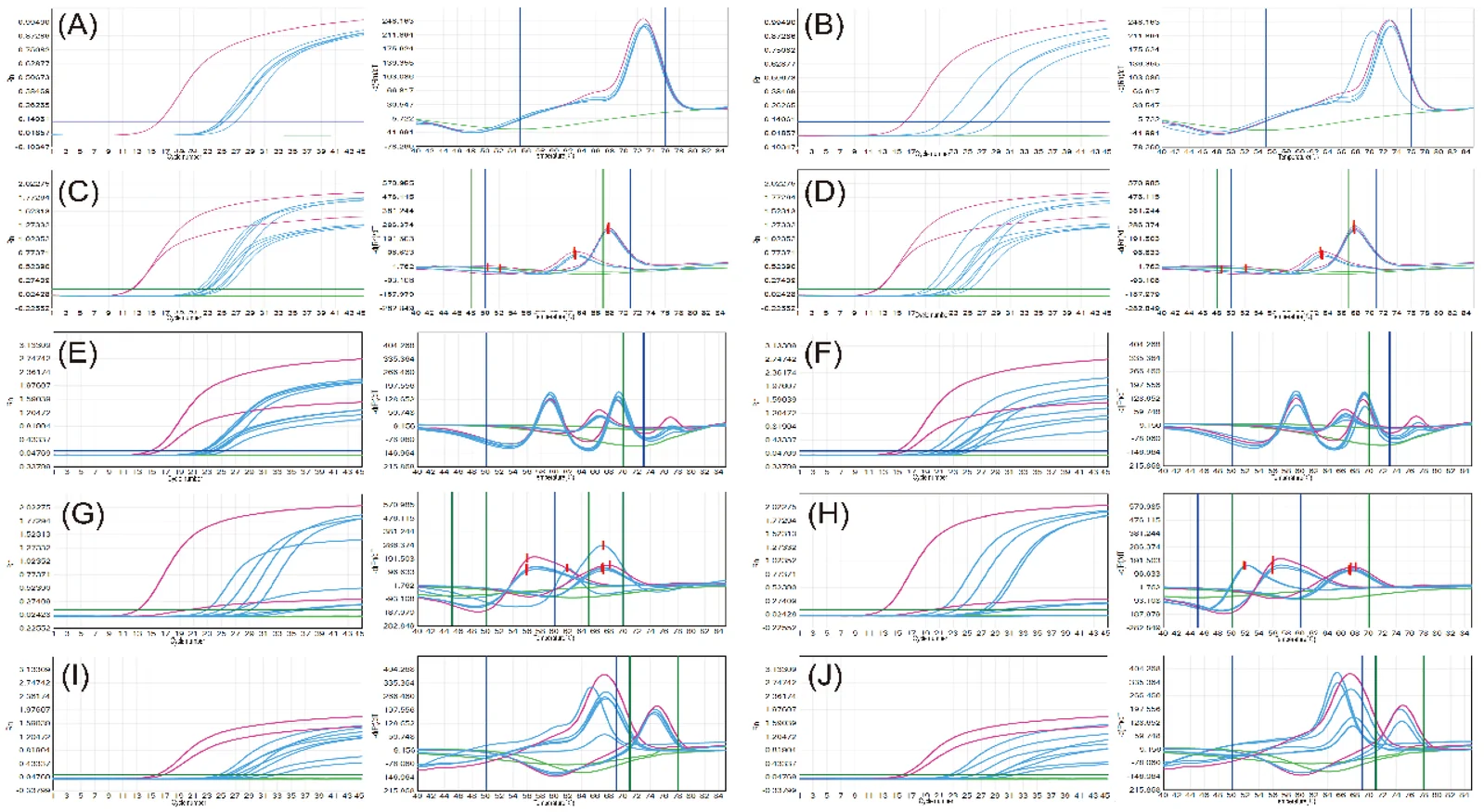
Comparison of drug resistance in CN-TB and PTB Mycobacterium tuberculosis. PCR and dissolution peak curves for quinolones, ethambutol, isoniazid, streptomycin, and rifampicin in nervous system tuberculosis **(A, C, E, G, I)**. PCR and dissolution peak curves for quinolones, ethambutol, isoniazid, streptomycin, and rifampicin in pulmonary tuberculosis **(B, D, F, H, J)**.

## 4 Discussion

CNS-TB is often associated with tuberculosis in multiple systems, including the lungs. This is because hematogenous dissemination is the primary mode of transmission for CNS-TB. In the early stages of infection, tubercle bacilli spread throughout the body via a low-level bacteremia. They form “Rich foci” in the brain and can also colonize other areas of the body (Noh et al., 2008; Rock et al., 2008). Evidence suggests that 40%–66% of adult patients with tuberculous meningitis (TBM) have extrameningeal tuberculosis at the time of diagnosis (Nelson and Zunt, 2011). This study also suggests that patients with CNS-TB often have tuberculosis involving multiple sites. Studies have shown that fever and loss of appetite are the most common clinical manifestations in patients with tuberculous meningitis (Srikanth et al., 2007). In this exploratory cohort, no statistically significant differences were observed between the CNS-TB and PTB groups in the prevalence of common systemic symptoms of active tuberculosis (fever, anorexia, weight loss and lymphadenopathy) or in the prevalence of dizziness. Studies by Naik S et al. have shown that both PTB and EPTB are associated with lymphocytopenia (Thwaites et al., 2000). However, in our exploratory cohort, patients with CNS-TB and PTB exhibited comparable hemoglobin levels and similar frequencies of abnormal lymphocyte counts, abnormal white blood cell counts, and abnormal absolute neutrophil counts. This discrepancy may be attributable to several factors, including differences in disease severity, extrapulmonary involvement, and the fact that two of our patients had already initiated anti-tuberculosis treatment prior to blood sampling—which could have partially restored hematological parameters toward normal ranges. Central nervous system tuberculosis has no distinct clinical or imaging features, resulting in a high rate of misdiagnosis (Tewary et al., 2005; Unal and Sutlas, 2005) The final diagnosis relies on pathological examination. Methods for obtaining pathological specimens include stereotactic biopsy and direct surgical resection. Stereotactic biopsy is only appropriate for diagnosing lesions that are more likely to be something other than tuberculomas, or for patients who cannot tolerate surgery. For patients whose intramedullary tumors remain unclear after MRI evaluation, or for those with progressively worsening neurological symptoms or increased intracranial pressure, aggressive brain biopsy or craniotomy for lesion removal is more appropriate. Etiological testing is the gold standard for diagnosing tuberculous disease of the nervous system. In tissue sections, Mycobacterium tuberculosis appears red under acid-fast staining. However, acid-fast staining is not very sensitive (Thwaites et al., 2000). Clinically, many patients with tuberculous tumors have negative results from both culture and acid-fast staining (Giese et al., 2003; Kilani et al., 2003; Mazodier et al., 2003). In our exploratory cohort, we did not detect a significant difference in acid-fast staining positivity between CNS-TB and PTB patients.The difference showed borderline statistical significance by Fisher’s exact test (P = 0.05, 95% CI: 0.005–0.760, which does not cross the null). This marginal result, combined with the limited power inherent in our small exploratory cohort, suggests caution in interpretation. Nevertheless, the large observed effect size indicates a potential group difference that merits further validation in an independent, larger sample set. However, molecular testing for Mtb was positive in all patients. Molecular testing is less likely to lead to missed diagnoses compared to acid-fast staining, highlighting its necessity.

The diagnostic criteria for tuberculosis based on pathological examination involve the detection of characteristic caseous necrosis. This includes inflammatory granulomas with caseous necrosis at their centers. Scattered lymphocytes, epithelial cells, and Langerhans cells can also be observed. The extent of the granulomas and the degree of necrosis vary. According to literature reports, the “macrophage center” hypothesis, which is based on the mononuclear macrophage system, divides the process of granuloma formation into three stages (van Furth et al., 1972; Tauber, 2003) First, monocytes infiltrate the tissue and mature into macrophages. Then, these mature macrophages aggregate to form granulomas. Finally, the macrophage granulomas evolve into epithelioid granulomas. The incidence of typical granulomas in CNS-TB lesions was no different from that in pulmonary tuberculosis lesions. Although there are significant immune differences between the central nervous system and the respiratory system, it’s possible that biopsies for CNS-TB are performed at a later stage, allowing sufficient time for granuloma formation in the affected areas. In terms of IHC, we observed significant differences in CD4^+^ T-cell infiltration between CNS-TB and PTB lesions. This may reflect divergent immune response magnitude and regulatory mechanisms in these two disease entities. CD4^+^ T cells play an important role in the protective immune response against tuberculosis infections (Raja, 2004; O'Garra et al., 2013). A significant reduction in CD4^+^ T cells due to human immunodeficiency virus infection increases the likelihood of new tuberculosis patients or reactivation of latent tuberculosis in patients (Bell and Noursadeghi, 2018). This is also supported by other studies.CD4^+^ T cells can be divided into several subgroups, including helper T cells(Th1, Th2, Th17) and regulatory T cells(Treg) (Wik and Skålhegg, 2022). Th2 can promote the production of antibodies like IgA by secreting cytokines such as IL-4. This helps prevent the spread of Mtb. Th2 cells also promote the polarization of macrophages into the M2 type, thereby facilitating the formation of granulomas. They also play a role in the immune response to TB (Gopal et al., 2014). IL-23, predominantly secreted by myeloid cells, drives Th17 cell differentiation. Th17 cells mainly produce IL-17 to recruit immune cells and amplify local anti-mycobacterial inflammation, rather than directly killing MTB (Lovey et al., 2022). Collectively, the IL-23/Th17 axis orchestrates inflammatory responses: Th17-derived IL-17 recruits neutrophils and other immune cells to infection sites and mediates tissue inflammation, serving as an amplifier of anti-mycobacterial immunity. Studies by Chen et al. showed that in patients with CNS-TB, not only is the absolute count of CD4^+^ T cells reduced, but also the counts of CD3^+^ and CD8^+^ T cells, as well as the overall lymphocyte count, are decreased (Chen et al., 2025). Although this exploratory finding of reduced CD4+ T cells in CNS-TB lesions relative to PTB lesions is preliminary, it is hypothesized that tissue-specific characteristics of the central nervous system may contribute to the altered immune cell infiltration observed in CNS-TB lesions. This speculative interpretation requires further verification. Further detailed characterization of immune cell subsets and their functional status will help to unravel the fundamental immunological mechanisms underlying tuberculosis.

The role of B cells in the tuberculosis immune response has not received much attention (Lu et al., 2016). However, some studies have shown that the glycosylation patterns of antibodies can help distinguish between latent and active tuberculosis, indicating that humoral immunity plays a certain role in controlling tuberculosis infections (Casadevall, 2016; Lu et al., 2016). This was also mentioned in studies by E. Kugelberg and others (Kugelberg, 2016). A paper by B.J. Hao Li also identified several possible immunological mechanisms by which antibodies exert their anti-tuberculosis effects (Li and Javid, 2018). CD38 is a multifunctional transmembrane protein that is expressed on the surfaces of various immune cells, including bone marrow progenitor cells, natural killer cells, monocytes, and activated T and B lymphocytes (Hartman et al., 2010).Silveira-Mattos et al. found in a cohort study of the Brazilian population that patients with active tuberculosis had a higher frequency of CD38^+^ expressing lymphocytes specific to tuberculosis compared to those with latent tuberculosis infection (Silveira-Mattos et al., 2020). CD38 immunohistochemistry, combined with morphological assessment, assists in identifying cells with plasma-cell features within tissue inflammatory infiltrates (Kumar et al., 2010). We observed a lower density of CD38^+^ cells with plasma-cell morphological features in CNS-TB lesions relative to PTB lesions. This finding suggests that this cell population may be less abundant within the local immune microenvironment of CNS-TB. B cells also exert non-negligible functions during tuberculous infection.

CD34 is mainly expressed by vascular endothelial cells, and its IHC signal can reflect lesion microvascular density to a certain extent. We observed higher CD34^+^ microvascular density in PTB lesions relative to CNS-TB lesions. This difference may stem from the distinct anatomical characteristics of lung and brain tissues, as well as divergent local host immune responses. We hypothesize that the blood-brain barrier may restrict vascular proliferation during Mtb infection, which could limit microvessel formation. Prior work has documented prominent VEGF-dependent angiogenesis within granulomas and perilesional tissue in active PTB (Golubinskaya et al., 2019). In contrast, the central nervous system is dominated by microglia. The blood-brain barrier constrains vascular proliferation during Mtb infection, potentially limiting microvessel formation. These interpretations remain preliminary given the small exploratory cohort and the absence of healthy tissue controls; further validation in larger patient series is required. In our experiments, the infiltration of CD56^+^ NK cells in CNS-TB was significantly higher than in PTB. It should be noted that our identification relies solely on CD56 staining. CD56 is not NK-cell specific in CNS tissue, and additional markers such as CD3 and NKp46 would be required to reliably identify true NK cells. A 2018 study suggested that latent tuberculosis is associated with cell-mediated killing, primarily mediated by CD16 and NK cells. The number of NK cells increased in the peripheral blood of individuals with latent tuberculosis, but decreased in patients with active tuberculosis. After recovery from tuberculosis, NK cell counts returned to their baseline levels (Roy Chowdhury et al., 2018). This descriptive observation provides a preliminary hypothesis for the differential local immune characteristics of CNS-TB lesions. The biological significance of altered CD56^+^ infiltrating immune cell abundance remains to be verified in further studies. In our exploratory cohort, CNS-TB lesions exhibited reduced CD4^+^ T-cell counts and CD38^+^ plasmacytoid cell counts, accompanied by relatively higher numbers of CD56^+^ lymphoid cells, compared with PTB lesions. However, given the cross-sectional and retrospective nature of this study, as well as the multiple potential confounders (including differences in tissue architecture, disease stage, prior anti-tuberculosis and corticosteroid treatment, and sampling sites), the functional interpretation of these observational differences remains uncertain. Whether the increased CD56^+^ lymphoid cell counts reflect a true local enrichment, a redistribution of immune subsets, or an artifact of tissue sampling cannot be determined from the current data. Future studies employing functional assays and larger, prospective cohorts are needed to elucidate the biological significance of these findings. If further validated in large-scale prospective studies and functional experiments, these preliminary descriptive findings may provide potential clues for understanding TB immunopathology and optimizing targeted immunotherapeutic strategies. In our exploratory analysis, no significant between-group differences were observed in the profiles of tuberculosis resistance-associated mutations. Although hematogenous dissemination of homologous mycobacterial clones represents a plausible biological mechanism, our small dataset is insufficient to support the direct extrapolation of pulmonary resistance profiles to guide clinical decision-making for CNS-TB. Further larger-scale studies are required to validate whether pulmonary resistance data can inform the management of CNS-TB and reduce adverse outcomes related to drug resistance.

## Data Availability

The raw data supporting the conclusions of this article will be made available by the authors, without undue reservation.
