# Supplementary material for: Comparative histopathological and immunohistochemical features of central nervous system and pulmonary tuberculosis: an exploratory retrospective study
Source: Front Cell Infect Microbiol. 2026 Oct 5;16:1933605. doi: 10.3389/fcimb.2026.1933605 (PMC13677463; doi:10.3389/fcimb.2026.1933605)
Supplement: Supplementary file 1 [file DataSheet1.pdf]

Supplementary table 1. Detailed information on CNS-TB cases.

Case	Clinical diagnosis	Disease duration / Operation date	Preoperative anti-tuberculosis treatment	Brain imaging findings	Lesion location	Sampling site	Surgical indication	Name of surgery
1	Tuberculous encephalitis	36 days / August 2014	None	Multiple abnormal signals in the right frontal lobe and brainstem	Right frontal lobe and brainstem	Right frontal lobe	Multiple intracranial lesions of unknown nature; no contraindications identified on preoperative examinations, and the patient was eligible for surgery.	Biopsy of intracranial lesion
2	Tuberculous meningitis	4 months / December 2, 2015	Yes, 13 days (prior anti-TB treatment 3 months earlier)	Combined with clinical history, findings were consistent with tuberculoma and tuberculous meningitis complicated with hydrocephalus; clinical correlation and comparison with previous images are recommended.	Cerebellum	Cerebellum	The patient was definitively diagnosed with hydrocephalus, increased intracranial pressure and tuberculous meningitis. Surgical indication was confirmed. No preoperative contraindications were noted.	Ventriculoperitoneal shunt + resection of cerebellar lesion
3	Cerebral tuberculoma, tuberculous meningitis	20 days / March 30, 2017	Yes, 2 days (prior anti-TB treatment 5 months earlier)	Findings consistent with tuberculous meningitis and multiple cerebral tuberculomas.	Left temporal lobe	Left temporal lobe	Space-occupying lesion in the left temporal lobe causing headache and memory decline; lesion nature remained unclear. Biopsy was indicated. Preoperative assessment was unremarkable with no obvious surgical contraindications.	Biopsy of intracranial lesion

Supplementary table 1. Detailed information on CNS-TB cases.								
Case	Clinical diagnosis	Disease duration / Operation date	Preoperative anti-tuberculosis treatment	Brain imaging findings	Lesion location	Sampling site	Surgical indication	Name of surgery
4	Multiple intracranial lesions	3 months / May 11, 2017	Yes, 14 days	Multiple scattered abnormal hyperdense lesions in the left cerebral hemisphere; clinical correlation and MRI correlation are recommended.	Scattered	Right frontotemporal region	Multiple intracranial lesions of unknown nature; no contraindications identified on preoperative examinations, and the patient was eligible for surgery.	Biopsy of intracranial lesion
5	Cerebral tuberculoma, pulmonary tuberculosis	2 months / January 18, 2018	Yes, 7 days (prior anti-TB treatment 1 month earlier)	Marked increase in intracranial nodular lesions.	Scattered	Right frontal region	Intracranial lesions of unknown nature; surgical biopsy was indicated. No preoperative contraindications were noted.	Biopsy of intracranial lesion
6	Tuberculous meningoencephalomyelitis, tuberculous meningitis, pulmonary tuberculosis	20 days / June 6, 2016	Yes, 14 days	Lesions involving brain parenchyma, meninges and spinal meninges suggestive of infectious disease, predominantly at the junction of the left frontal, parietal, occipital and temporal lobes. Clinical correlation is recommended.	Scattered	Left parietal lobe	Unclear diagnosis limiting further therapeutic planning; no contraindications identified on preoperative examinations, and the patient was eligible for surgery.	Craniotomy for biopsy

Supplementary table 1. Detailed information on CNS-TB cases.

Case	Clinical diagnosis	Disease duration / Operation date	Preoperative anti-tuberculosis treatment	Brain imaging findings	Lesion location	Sampling site	Surgical indication	Name of surgery
7	Intracranial cerebral tuberculoma, tuberculous brain abscess, abdominal tuberculosis, intestinal tuberculosis, pulmonary tuberculosis	10 days / January 6, 2022	Yes, 13 days (intermittent anti-TB treatment over 20 years; most recent course 3 months prior)	1. Lesion with enhancement and surrounding edema at the left frontoparietal junction, suggestive of infectious lesion (tuberculoma favored, metastasis to be excluded). 2. Small ischemic foci in the brain.	Left frontoparietal junction	Left frontoparietal junction	The patient presented with acute right limb weakness. Contrast-enhanced brain MRI highly suggested cerebral tuberculoma and tuberculous brain abscess. No preoperative contraindications were noted.	Resection of intracranial tuberculoma and tuberculous brain abscess
8	Cerebral tuberculoma	5 months / September 18, 2024	None	1. Multiple abnormally enhanced lesions in the left frontal lobe with surrounding brain edema and adjacent meningeal involvement; lymphoma was suspected and differential diagnoses remained open. 2. Subfalcine hernia and partial empty sella.	Left frontal lobe	Left frontal lobe	Defined space-occupying lesion in the left frontal lobe with marked perilesional edema and rightward midline shift. No preoperative contraindications were noted.	Resection of giant deep-seated lesion in left frontoparietal lobe

Supplementary table 1. Detailed information on CNS-TB cases.

Case	Clinical diagnosis	Disease duration / Operation date	Preoperative anti-tuberculosis treatment	Brain imaging findings	Lesion location	Sampling site	Surgical indication	Name of surgery
9	Tuberculoma, tuberculous meningitis	1 year / December 6, 2024	Yes, 9 days (prior anti-TB treatment 1 year earlier)	Multiple enhanced intracranial lesions with partial meningeal enhancement, suggestive of tuberculous meningitis and tuberculoma.	Right frontal lobe	Right frontal lobe	Multiple intracranial lesions highly suspicious for tuberculoma, without pathological confirmation. Surgery was indicated, and no absolute preoperative contraindications were identified.	Stereotactic-guided resection and biopsy of right frontal lesion

Supplementary table 2. CNS-TB and PTB immune cell counts raw quantitative data.

Case	Sex	Age (year)	GFAP (cells/HPF)	Ki-67	S-100 (cells/HPF)	CD3 (cells/HPF)	CD4 (cells/HPF)	CD8 (cells/HPF)	CD20 (cells/HPF)	CD38 (cells/HPF)	CD34 (vessels/HPF)	CD56 (cells/HPF)	CD68 (cells/HPF)	LCA (cells/HPF)	PD-1 (cells/HPF)	PD-L1 (cells/HPF)
1	M	49	0	<2%	80	251	10	180	90	0	9	0	208	40	0	0
2	F	33	1	<5%	11	172	66	131	80	8	6	0	28	86	3	30
3	M	27	25	<2%	207	14	5	9	1	1	9	490	129	10	30	20
4	F	28	2	<20%	40	289	141	145	14	6	11	11	180	84	0	100
5	M	49	0	<2%	1	16	4	12	5	1	0	1	2	20	0	0
6	F	45	1	<10%	15	152	91	61	28	0	9	7	138	27	0	65
7	M	45	1	<5%	15	218	88	129	39	26	5	1	33	107	0	59
8	F	47	0	<20%	8	274	152	122	4	19	20	12	57	80	0	165
9	F	48	0	<25%	16	525	362	164	46	30	16	3	8	203	0	187
10	F	52	0	<10%	85	775	509	266	63	15	48	1	168	153	0	119
11	M	43	0	<5%	1	388	252	136	17	89	22	0	107	144	0	152
12	M	52	0	<10%	18	140	100	104	42	43	13	1	102	44	0	107
13	M	53	0	<20%	10	348	223	125	121	81	27	1	137	80	0	145
14	M	66	0	<5%	43	367	236	129	33	29	6	0	82	14	1	40
15	M	75	0	<10%	5	448	252	196	380	68	16	0	57	97	0	49
16	M	49	0	<10%	20	844	496	348	244	52	14	0	188	194	0	115
17	F	62	0	<5%	7	202	101	100	44	41	21	0	52	63	0	47
18	M	66	0	<5%	19	104	60	44	33	53	35	0	11	33	2	9

Supplementary table 3. Specific details of the case and time of blood draw.

Case	Sex/Age (years)	Disease duration	Operation date	Preoperative anti-TB treatment	Corticosteroid use
1	M/49	36 days	Aug 12, 2014	No	Yes (before corticosteroid)
2	F/33	4 months	Dec 2, 2015	Yes (before anti-TB drugs)	Yes (before corticosteroid)
3	M/27	20 days	Mar 30, 2017	Yes (before anti-TB drugs)	No
4	F/28	3 months	May 11, 2017	Yes (before anti-TB drugs)	No
5	M/49	2 months	Jan 18, 2018	Yes (before anti-TB drugs)	Yes (before corticosteroid)
6	F/45	20 days	Jun 6, 2016	Yes (before anti-TB drugs)	No
7	M/45	10 days	Jan 6, 2022	Yes (intermittent anti-TB in past 20 years)	No
8	F/47	5 months	Sep 18, 2024	No	Yes (before corticosteroid)
9	F/48	1 year	Dec 6, 2024	Yes	Yes (before corticosteroid)
10	F/52	1 year	Sep 25, 2024	No	No
11	M/43	3 months	Dec 18, 2024	No	No
12	M/52	2 weeks	Jan 24, 2025	No	No
13	M/53	8 days	Jan 10, 2025	No	No
14	M/66	2 months	Feb 24, 2025	No	No
15	M/75	6 months	Mar 24, 2025	No	No
16	M/49	1 week	Mar 23, 2023	No	No
17	F/62	1 month	Jun 18, 2024	No	No
18	M/66	2 weeks	Aug 6, 2024	No	No

Supplementary table 4. Specific values from the complete blood count.

Case	Hb (g/L)<br>Reference range:<br>Males:120–160 g/L<br>Females:110–150 g/L	WBC (×10⁹/L)<br>Reference range:<br>3.5–9.5×10⁹/L	Neutrophil (×10⁹/L)<br>Reference range:<br>2.0–7.5×10⁹/L	Lymphocyte (×10⁹/L)<br>Reference range:<br>0.8–4.0×10⁹/L
1	140	13.79	12.88	0.58
2	114	6.20	4.60	0.97
3	151	8.43	5.58	2.20
4	131	7.80	6.05	1.04
5	126	5.70	4.15	0.96
6	134	4.74	3.07	0.92
7	136	6.75	5.65	0.85
8	107	5.53	3.63	1.36
9	97	7.42	4.45	2.30
10	112	5.35	3.01	1.50
11	166	7.70	4.44	2.58
12	140	5.31	2.53	2.18
13	160	6.63	4.31	1.60
14	158	4.08	1.62	1.66
15	149	6.35	3.55	2.13
16	159	6.51	3.91	2.11
17	133	6.11	4.07	1.64
18	124	4.14	2.40	1.22
